# Soy isoflavone extracts stimulate the growth of nude mouse xenografts bearing estrogen-dependent human breast cancer cells (MCF-7)^☆^

**DOI:** 10.1016/S1674-8301(12)60006-2

**Published:** 2012-01

**Authors:** Qian Wu, Ye Yang, Jing Yu, Nianzu Jin

**Affiliations:** Department of Hygienic Analysis and Detection, School of Public Health, Nanjing Medical University, 140 Hanzhong Road, Nanjing, Jiangsu 210029, China.

**Keywords:** soy isoflavone extracts, breast cancer, nude mice, MCF-7, estrogen, ki-67, pS2

## Abstract

We explored the effects of different lifetime exposures to soy isoflavone extracts on the growth of estrogen-dependent human breast cancer cells (MCF-7) implanted into athymic mice of different ovarian statuses. The athymic mice, ovariectomized or not, were implanted with MCF-7 cells. Mice were fed with low, moderate and high doses of soy isoflavone extract, at dietary concentrations of 6.25, 12.5 and 25 g/kg, in different reproductive models, respectively. The expression of ki-67 was detected by immunohistochemistry. pS_2_ expression in tumors was analyzed by real-time PCR. Estrogen level in the serum was measured by chemiluminescence enzyme immunoassay. Total genistein and daidzein levels in serum and urine were determined by liquid chromatography-electrospray tandem mass spectrometry (LC-ES/MS/MS). In Group A, on week 4, nude mice were exposed to different doses of soy iosflavone extracts. In Group B, the experimental diets were given to the nude mice following ovariectomy and tumor implantation. In both groups, 6.25 and 12.5 g/kg soy isoflavone extracts stimulated the growth of MCF-7 xenografts, increased pS_2_ expression, proliferation and estrogen level in serum. In both Group B (postmenopausal mouse model) and Group C (premenopausal mouse model), soy isoflavone extracts at doses of 6.25 and 12.5 g/kg showed stimulatory effects on the growth of MCF-7 tumors. In conclusion, administration of soy isoflavone extracts at doses of 6.25 and 12.5 g/kg during adolescence or later in life stimulated tumor growth in both menopausal and postmenopausal mouse models.

## INTRODUCTION

Soy-based food is consumed in significantly high quantities by Asian women, and high soy food intake may contribute to cancer protection[1]. Soy contains large amounts of bioactive substances, which have been related to the growth of breast cancer, and soy isoflavones have attracted the most attention. Many studies have been conducted to elucidate the effects of soy isoflavones on breast cancer. Isoflavones act as estrogen agonists by binding to the estrogen receptor and generating estrogen-induced responses.

Epidemiological studies have suggested that the intake of soy food may reduce the occurrence of breast cancer due to isoflavones, especially in China and Japan. For example, tofu and miso have been shown to have distinct effects on preventing breast cancer. Trock *et al*[2]. performed a meta analysis of 10 epidemiological studies that were stratified by menopausal status. The inverse association between soy exposure and breast cancer risk was somewhat stronger in premenopausal women than in postmenopausal women. Seventy-five percent of new breast cancer cases are diagnosed in postmenopausal women (> 50 years of age)[3]. However, high doses of soy isoflavone supplementation, such as soy protein isolates or isoflavone capsules have been recommended to prevent the occurrence of breast cancer. In addition, early life exposure may influence the endocrine hormone level, and then increase the risk of breast cancer. Breast tissue undergoes substantial cellular proliferation during adolescence[4]. Adolescent mammary tissue is therefore probably more sensitive to carcinogenic insult than adult mammary tissue. It has been reported that high soy food intake during adolescence may reduce the subsequent risk of breast cancer in later life[5],[6].

However, results from the *in vivo* animal tests were quite contradictory. At different pharmacological and physical concentrations and at different life stages, soy isoflavone may exhibit stimulatory as well as inhibitory effects *in vivo* and *in vitro*, but the mechanisms of action are still unclear. Since *in vivo* and *in vitro* data are not consistent with epidemiological investigations, the association between soy isoflavone and breast cancer risk should be rigorously evaluated before safe and reliable recommendations are concluded.

Single components, e.g., genistein, daidzein or equol, are usually used in the reported experimental studies of the association between soy isoflavone and breast cancer risk. We hypothesized that there might be some *in vivo* differences in metabolism between single component and total extracts of isoflavone, or there might be a synergistic or antagonistic effect among various components. In this study, we added commercially available total extracts of soy isoflavones to animal diets to investigate the effects, during different life stages, of exposure to soy isoflavone extracts on the growth of estrogen dependent human breast cancer *in vivo*.

## MATERIALS AND METHODS

### Materials

Twenty-four female, 3-week-old athymic nude mice (BALB/c-nu/nu) were purchased from Shanghai SLAC Laboratory Animal Co., Ltd., China, each weighing 16.0–18.0 g, and bred in laminar flow hood using plastic cages with filter caps. Estrogen receptor-positive human breast cancer cells (MCF-7), were a gift from Prof. Yujie Sun, School of Basic Medical Sciences, Nanjing Medical University, China. Dulbecco's Modified Eagle Medium (DMEM), Gibco, USA); trypsin (Sigma, USA); bovine calf serum (BCS, Hangzhou Sijiqing Biological Engineering Materials Co., Ltd., China); soy isoflavone extracts (Shanghai Fanghua Biotech Co., Ltd., China; components analyzed by HPLC, genistin: 25.78%, genistein: 0.62%, daidzin: 9.39%, and daidzein: 1.20%; estradiol (E_2_) patch (Zhejiang Yatai Pharmaceutical Co., Ltd., China); Ki-67 immunohistochemical kits (Wuhan Boster Biological Technology., Ltd., China); total RNA extraction kit (Shanghai Shenergy Biocolor BioScience & Technology Co., Shanghai, China); real-time PCR reagents [TAKARA Biotechnology (Dalian) Co., Ltd., Dalian, China]; animal diets (Jiangsu Xietong Pharmaceutical Biotechnology Co., Ltd., China).

### Tumor growth model

**Fig. 1:**
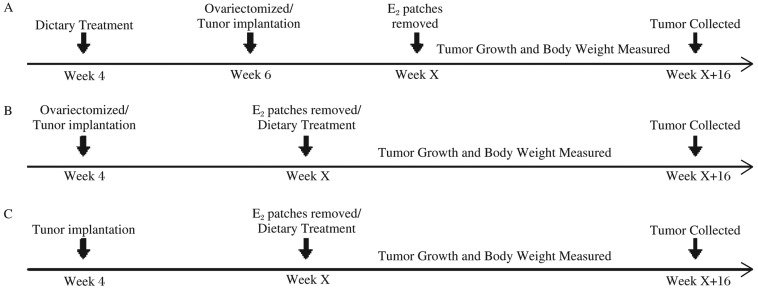
The mammary tumor study flowchart. E_2_ patches were used to stimulate mammary tumor growth after injection of MCF-7 cells. Each group was treated with different doses of soy isoflavone extracts in different lifetimes. Tumor growth and body weight were measured weekly. After treatment for 16 weeks, the mice were sacrificed and the blood/tissues were collected. A: Group A; B: Group B; C: Group C.

Three-week old female athymic nude mice were acclimated for one week, and then were randomly assigned to three groups (***Fig. 1***). Mice in Group A were fed with the diet containing different doses of soy isoflavone extracts (at dietary concentrations of 0, 6.25, 12.5 and 25 g/kg, using genistein as standard) for two weeks, and then ovariectomized. After recovery, E_2_ patches (containing 0.6 mg estradiol) were placed on their backs for 7 d. One week later, MCF-7 cells were injected at 200 µL (1×10^7^ cells/200 µL) per site into the flanks of nude mice. The E_2_ patches were removed until the tumor surface area reached 10 mm^2^. Mice in Group B were first ovariectomized and then implanted with MCF-7 cells using the aforementioned method. The mice were fed with the diet containing different doses of isoflavone extracts (at dietary concentrations of 6.25, 12.5 and 25 g/kg) until the tumor surface area reached 10 mm^2^. Mice in Group C were first implanted with MCF-7 cells and then fed with the diet containing different doses of soy isoflavone extracts (at dietary concentrations of 0, 6.25, 12.5 and 25 g/kg). On week 16 post treatment, all mice were sacrificed and the blood/tissues were collected.

### Tumor implantation

MCF-7 cells were cultured in DMEM containing 10% BCS, 100 µg/mL penicillin, 100 U/ml streptomycin, 0.2 U/mL insulin and 0.1 nmol/L 17β-estradiol. MCF-7 cells were then maintained at 37°C under a 5% CO_2_ atmosphere. Cells were harvested using trypsin-EDTA, and injected at 200 µL (1×10^7^ cells/200 µL) per site into the flanks of nude mice.

The tumor growth and body weight were measured weekly for 16 weeks and the surface area was calculated using the formula [(length/2+width/2)π]. All animal procedures were approved by the Experimental Animal Care and Use Committee of Nanjing Medical University.

### Tumor/blood collection

At the end of the study, mice were sacrificed by cervical dislocation and the tumor and blood samples were collected. Tumors from each mouse were snap frozen by submersion in liquid nitrogen for immunohistochemical staining and RNA isolation. Blood from eyeball vein was centrifuged at 1,200 *g* for 5 min, and then the serum was obtained, which was stored at 4°C for analysis.

### The expression of ki-67 detected by immunohistochemistry

The mice were sacrificed after blood sampling. The tumors were removed quickly, some were stored in liquid nitrogen for RNA isolation, and some were fixed in 10% neutral formalin, embedded in paraffin, and cut into 4 µm sections. The slides were deparaffinized by immersing in xylene and hydrated by immersing in gradient ethanol. To block endogenous peroxidase, the slides were incubated with 3% H_2_O_2_ for 5-10 min, and then the slides were washed with PBS for 2 min three times. Mouse anti-human Ki-67 antibody (1:3,000, Zhongshan Company, Beijing, China) was added to the slides, which were then incubated at 37°C for 1-2 h. After the slides were washed with PBS, the HRP-labeled goat anti-mouse IgG antibody (Zhongshan Company, Beijing, China) was added to the slides and incubated at 37°C for 30 min. Slides were then washed with PBS. DAB was added to each slide. Slides were then washed twice in PBS, counterstained with hematoxlin, and then dehydrated by gradient ethanol followed by clearing in xylene. Slides were mounted and analyzed using a light microscope (400×) by two senior pathologists. A total of 25 fields from 5 tumors per treatment group were evaluated. Both positive and background stained cells were counted. The data were then presented as a percentage of cell proliferation.

### The expression of pS_2_ detected by real-time PCR

Tumor tissue samples of 100 mg were homogenized with 1 mL Trizol on the ice. Total RNA was extracted according to the instruction of the kit. cDNA was synthesized from the total RNA (2 µg) using random hexamer primers and PrimeScriptTM RTase. Real-time PCR was run on Chromo4 Four-color Real-time system (BIO-RAD, USA) using SYBR Premix Ex Taq^TM^. Each sample had three parallel samples, and GAPDH was selected as the internal control. The gene levels were determined with the double delta method (2^−ΔΔCt^). Primer sequences: pS2, F: 5′-AATAAGGGCTGCTGTTTCG-3′', R: 5′-AAGCGTGTCTGAGGTGTCC-3′; GAPDH, F: 5′-GAAGGTGAAGGTCGGAGTC-3′', R: 5′-GAAGATGGTGATGGGATTTC-3′.

### Estrogen level in serum detected by chemiluminescence enzyme immunoassay

Blood sample from eyeball vein of mice was centrifuged at 1,200 *g* for 5 min, and then the serum was obtained. E_2_ level was detected using the Beckman Coulter Access analyzer.

### Serum and urine analysis

Serum and urine samples were analyzed using isotope dilution liquid chromatography-electrospray tandem mass spectrometry (LC-ES/MS/MS) described previously[7],[8]. Levels of total genstein and daidzein were determined in 100 µL sera post treatment with β-glucuronidase/sulfatase for deconjugation and phenytoin was selected as the internal standard. Five to eight serum samples per treatment group were analyzed. The limits of detection for the two analytes were approximately 200 pg/mL, the accuracy of the intra-day method was 85.05%-114.67%, and the precision of the method was 2.9%-11.3%[7].

### Statistical analysis

The data were presented as mean±standard deviation (SD). The significance of the effect caused by the soy isoflavone extracts was determined by Mann-Whitney, Kruskal-Wallis or χ^2^ test. All *P*-values were two-sided and those less than 0.05 were considered statistically significant. All statistical analyses were performed using version 17.0 of the SPSS software package (SPSS Inc., Chicago, IL, USA).

## RESULTS

### Tumor growth

**Fig. 2:**
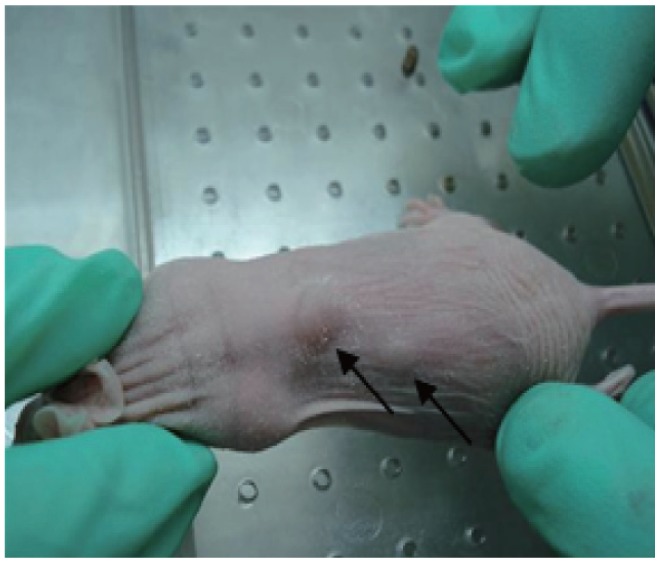
The implanted tumor in athymic mice. The tumor appeared on d 5 after injection. Tumor xenograte at d 30 was shown here and indicated by arrows.

Five days after injection of MCF-7 cells, tumor nodule appeared hard at the injected site. When the tumor surface area reached 10 mm^2^, the E_2_ patches were removed[9],[10] (***Fig. 2***).

**Fig. 3:**
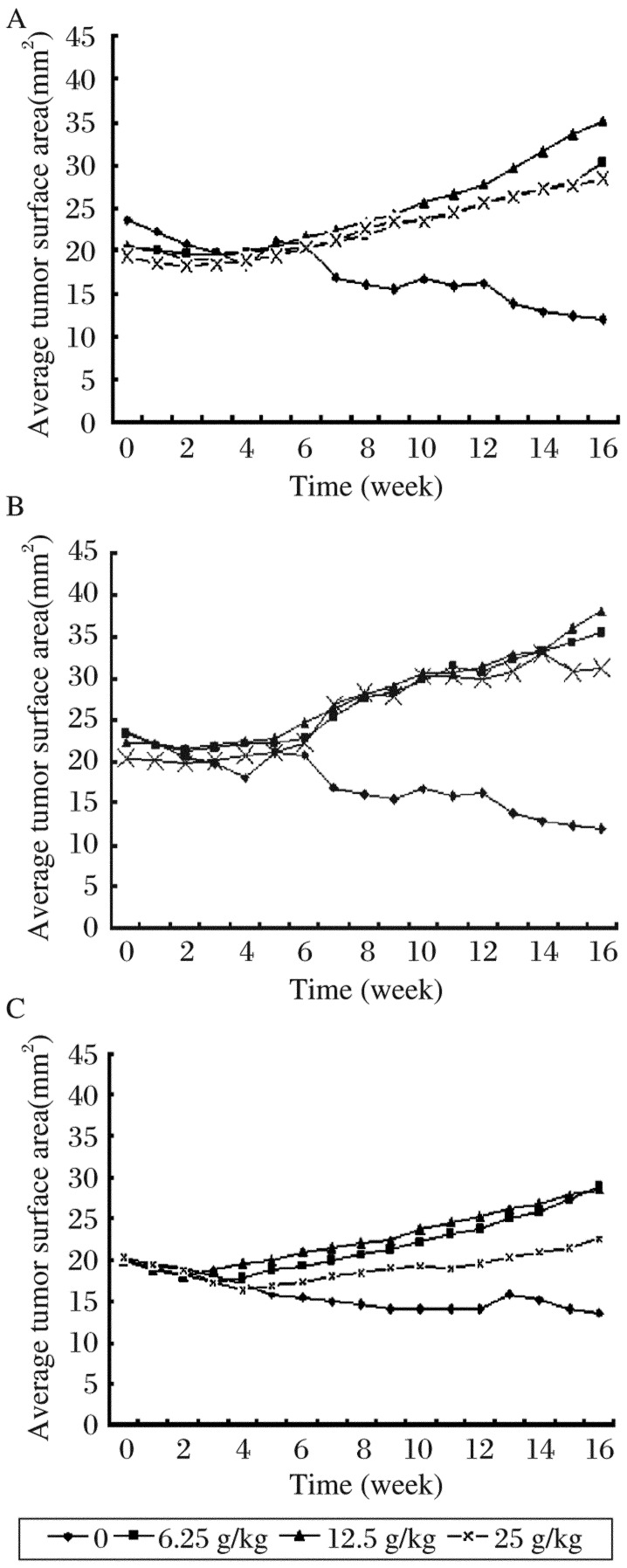
Effects of soy isoflavone on MCF-7 tumor growth in nude mice. Mice were assigned into three treatment groups: Group A (A), Group B (B), and Group C (C). Week 0 was the first day that animals were started on the experimental diets. The tumors were measured weekly. The surface area was calculated according to the length and width. The results shown were the average mean of eight mice for each group (mm^2^).

As shown in ***Fig. 2***, the mean tumor surface areas in each group were monitored for 16 w. Because of the removal of E_2_ patches, the tumors in each group had no obvious changes in the earlier six weeks. Then, the tumors in the treatment groups appeared to grow slowly over time, but steady regression in tumors were observed in the control group. At the end of the study, the mean tumor surface areas of each group were as follows: Group A: (11.97±2.47), (30.2±1.01), (35.17±2.65), and (28.4±1.45) mm^2^ for mice receiving 0, 6.25, 12.5 and 25 g/kg soy isoflavone extracts, respectively; Group B: (11.97±2.47), (35.5±1.93), (38.13±0.55), and (31.28±3.78) mm^2^ for mice receiving 0, 6.25, 12.5 and 25 g/kg soy isoflavone extracts, respectively; Group C: (13.63±1.62) mm^2^, (28.8±2.2) mm^2^, 28.5±2.9, and (22.56±2.45) mm^2^ for mice receiving 0, 6.25, 12.5 and 25 g/kg soy isoflavone extracts, respectively. In groups A, B and C, there were statistically significant differences in tumor areas between the control and all the treatment groups, respectively (*P* < 0.05, ***Fig. 3***).

### Cell proliferation in tumors

**Fig. 4:**
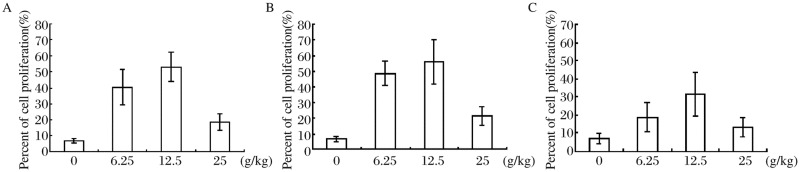
Effect of soy isoflavone extracts on proliferation of MCF-7 cells. Ki-67 expression was evaluated as an immunohistochemical marker for proliferation. A total of 25 fields from 5 tumors per treatment group were evaluated. The percentage of cell proliferation represented the positive cells in the given area of tissue. A: cell proliferation in Group A; B: cell proliferation in Group B; C: cell proliferation in Group C.

Cell proliferation in tumors was determined using immunohistochemistry. The percentages of ki-67 expression in Group A were (6.7±1.5)%, (40.3±11.0)%, (53.3±9.1)%, (18.5±5.1)% for mice receiving 0, 6.25, 12.5 and 25 g/kg soy isoflavone extracts, respectively. The expression of ki-67 increased in mice receiving 6.25 and 12.5 g/kg soy isoflavone extracts, and a significant difference was observed in comparison with the control (*P* < 0.05), but no significant difference was detected when compared with mice receiving 25 g/kg soy isoflavone extracts (*P* > 0.05). The percentages of ki-67 expression in Group B were (6.7±1.5)%, (48.5±7.9)%, (56.0±14.2)%, and (21.4±6.1)% for mice receiving 0, 6.25, 12.5 and 25 g/kg soy isoflavone extracts, respectively. Statistically significant differences in the expression of ki-67 were observed in mice receiving 6.25 and 12.5 g/kg soy isoflavone extracts compared with that of the control (*P* < 0.05), but no significant difference was detected when compared with mice receiving 25 g/kg soy isoflavone extracts (*P* > 0.05). The percentage of ki-67 expression in Group C was (7.0±2.7)%, (18.8±8.3)%, (31.7±12.1)% and (13.2±5.1)% for mice receiving 0, 6.25, 12.5 and 25 g/kg soy isoflavone extracts, respectively. There were statistically significant differences in the expression of ki-67 in mice receiving 6.25 and 12.5 g/kg soy isoflavone extracts compared with that of the control (*P* < 0.05), but no significant difference was detected when compared with mice receiving 25 g/kg soy isoflavone extracts (***Fig. 4***).

### pS_2_ expression in tumors

**Fig. 5:**
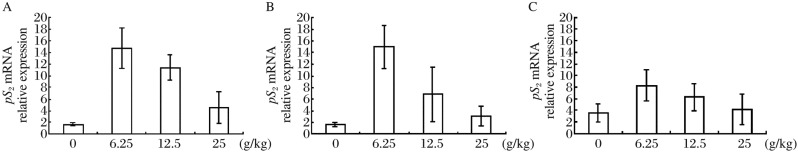
Effect of soy isoflavone extracts on *pS_2_* expression. *pS_2_* expression was analyzed using real-time PCR and GADPH was selected as internal control. Numbers on the y axis represent the relative mRNA level. A: Group A; B: Group B; C: Group C.

To evaluate the effect of soy isoflavone extracts on the expression of estrogen-responsive gene, the expression of *pS_2_* was measured using real-time PCR. In Group A, pS_2_ expression was significantly increased in mice receiving 6.25, 12.5 and 25 g/kg of the soy isoflavone extracts compared with the vehicle control group (all *P* values < 0.05). In Group B, pS_2_ expression was increased in mice receiving 6.25 (*P* < 0.05), 12.5 (*P* < 0.05) and 25 g/kg (*P* > 0.05) soy isoflavone extracts compared with the vehicle control group. In Group C, pS_2_ expression was increased by 6.25 (*P* < 0.05), 12.5 (*P* > 0.05) and 25 g/kg (*P* > 0.05) soy isoflavone extracts compared with the vehicle control group (***Fig. 5***).

### Serum estrogen levels

**Fig. 6:**
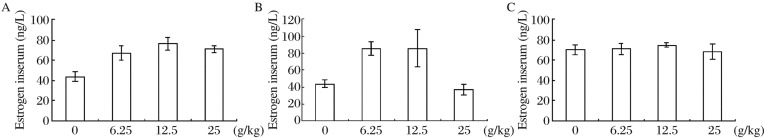
The concentration of estrogen in serum in groups A, B and C. Estrogen serum levels were determined by chemiluminescence immunoassay. A: Group A; B: Group B; C: Group C.

The concentrations of estrogen in serum in Group A were (44.0±4.58), (67.13±7.16), (76.2±6.45) and (71.0±3.58) ng/L in mice receiving 0, 6.25, 12.5 and 25 g/kg soy isoflavone extracts, respectively. In Group A, the estrogen level was significantly increased by soy isoflavone extracts (*P* < 0.05). The concentrations of estrogen in serum in Group B were (44.0±4.58), (85.5±7.66), (85.75±21.75) and (37±6.35) ng/L in mice receiving 0, 6.25, 12.5 and 25 g/kg soy isoflavone extracts, respectively. In Group B, estrogen level was significantly increased in mice receiving 6.25 and 12.5 g/kg soy isoflavone extracts (*P* < 0.05), but not in mice receiving 25 g/kg soy isoflavone extracts (*P* > 0.05). The concentrations of estrogen in serum in Group C were (70.13±4.82), (70.63±6.05), (74.55±2.52) and (68.5±7.41) ng/L in mice receiving 0, 6.25, 12.5 and 25 g/kg soy isoflavone extracts, respectively. There were no statistically significant differences among mice receiving different doses of soy isoflavone extracts in Group C (***Fig. 6***).

### Isoflavone levels in serum and urine

The concentrations of total genistein and daidzein in serum and urine from mice fed with isoflavone extracts nude mice increased regularly as the dietary dose increased in the three groups A, B and C (***Table 1***).

**Table 1:** Genistein and daidzein levels in serum and urine

Group	Dose (g/kg)	Genistein(ng/mL)		Daidzein(ng/mL)	
		Serum	Urine	Serum	Urine
Group A	0	85 ± 166	4457 ± 6329	751 ± 751	79443 ± 130673
	6.25	24 ± 69	22230 ± 26478	243 ± 628	175729 ± 155494
	12.5	102 ± 58	50531 ± 28552	129 ± 129	544734 ± 288620
	25.0	1400 ± 1141	55053 ± 21808	3843 ± 3843	718022 ± 106312
Group B	0	0	0	0	0
	6.25	54 ± 47	63900 ± 17600	827 ± 530	744932 ± 97879
	12.5	73 ± 41	108421 ± 42254	1525 ±1222	890793 ± 292855
	25.0	105 ± 105	327938 ± 391396	1653 ± 594	1210856 ± 1132913
Group C	0	0	0	0	0
	6.25	231 ± 487	41698 ± 27517	1682 ± 1867	821262 ± 144547
	12.5	604 ± 787	150914 ± 38031	2688 ± 2013	902685 ± 183485
	25.0	2406 ± 1161	318376 ± 117520	5425 ± 1501	1345788 ± 323073

Group A: Mice were fed with the diet containing different doses of soy isoflavone extracts (at dietary concentrations of 0, 6.25, 12.5 and 25 g/kg, using genistein as standard) for two weeks, and then ovariectomized. After recovery, E2 patches (containing 0.6 mg estradiol) were placed on their backs for 7 d. One week later, MCF-7 cells were injected at 200 (µL (1×10^7^ cells/200 (µL) per site into the flanks of nude mice. The E2 patches were removed until the tumor surface area reached 10 mm^2^.

Group B: Mice were first ovariectomized and then implanted with MCF-7 cells using the aforementioned method. The mice were fed with the diet containing different doses of isoflavone extracts (at dietary concentrations of 6.25, 12.5 and 25 g/kg) until the tumor surface area reached 10 mm^2^. Group C: Mice were first implanted with MCF-7 cells and then fed with the diet containing different doses of soy isoflavone extracts (at dietary concentrations of 0, 6.25,12.5 and 25 g/kg).

(mean±SD)

## DISCUSSION

Soy contains large amounts of isoflavone, genistin and daidzin, which are metabolized to genistein and daidzein by gut microflora[1],[11]. Genistein has many biological effects that could influence breast cancer risk. For example, genistein exhibits estrogenic properties at a physical concentration (0.5-5 µmol/L)[12], and some effects that could enhance the growth of human breast xenografts[13]. Daidzein, another key isoflavone, promoted the growth of MCF-7 cells *in vitro* at 0.001-50 µmol/L, and exerted a slight but significant stimulatory effect on tumor growth at 1000 ppm dietary daidzein in nude mouse model[14]. The total extracts of isoflavone employed in this study are commercially available, for use in healthy food. The contents of soy isoflavone extracts include 25.78% genistin, 0.62% genistein, 9.39% daidzin, and 1.20% daidzein. In view of the inconsistent results of genistein and daidzein, it is hypothesized that there might be some differences in estrogenic effects *in vivo* between the single component[12]-[14] and total soy isoflavone extracts.

First, we examined the different exposure times of soy iosflavone extracts. In Group A, on week 4 nude mice were exposed to different doses of soy iosflavone extracts. In Group B, nude mice were exposed to soy iosflavone extracts after they were ovariectomized and tumor was implanted. In both groups, administration at doses of 6.25 and 12.5 g/kg stimulated the growth of MCF-7 xenograft and increased pS2 expression, proliferation and estrogen level in serum. Although the stimulatory effect in Group B was slightly higher than that of Group A, there was no statistical significance (*P* > 0.05). Prepubertal genistein exposure in rats suppressed mammary tumors that formed after the subsequent exposure to a chemical carcinogen[15]-[17]. In different animal models, genistien may affect the development of breast cancer or tumor formation in a different way. Meanwhile, the effects of soy isoflavone extracts in premenopausal and postmenopausal mouse models were observed. The ovarian status may partly explain the different effects of soy on breast cancer risk in populations[2]. But in our studies, in either Group B (postmenopausal mouse model) or Group C (premenopausal mouse model), soy isoflavone extracts at doses of 6.25 and 12.5 g/kg showed stimulatory effects on the growth of MCF-7 tumors. In Group C, no difference was found in the estrogenic levels among the 4 subgroups, which may be due to the ovarian status. Another study also showed that genistein stimulated the growth of chemically induced mammary tumors in ovariectomized rats[18]. The effects of 25 g/kg of soy isoflavone extracts in groups A, B and C were complicated. It may be that the concentration of isoflavone in the serum did not increase with increased supplement dose because of the metabolism of isoflavone *in vivo*. This study provided evidence that soy isoflavone extracts could stimulate the growth of MCF-7 cells *in vivo* at different exposure times and in different ovarian statuses, which was partly similar to other studies reporting the effects of genistien on stimulating breast cancer xenograft growth in ovariectomized mice[13],[14],[19]. As indicated, genistein was the main component of soybean isoflavone that exhibited estrogenic properties, and its effect was not influenced by other components.

Genistein and other metabolites can bind to the estrogen receptors (ERα and ERβ), serving as a natural supplement of estrogen to regulate the hormonal level. The plasma concentration of endogenous estrogen is very important for mammary development and will vary considerably through the lifespan of women, which will lead to the different effects of soy isoflavone on mammary gland at different levels of estrogen. Our study showed that estrogen levels at doses of 6.25 and 12.5 g/kg were significantly higher than those of the control group, which indicated that soy isoflavone had a weak estrogenic effect on upregulating estrogen level when estrogen level was low in the blood (such as ovariectomized). As deduced from these results, soy isoflavone is particularly useful in relieving menopausal symptoms. However, soy isoflavone at doses of 6.25 and 12.5 g/kg induced cell proliferation in tumors and promoted pS_2_ expression, which suggested that soy isoflavone in the tested concentration range stimulated the growth of tumors in ovariectomized nude mice. Therefore, intake of highly refined or higher doses of soy isoflavone may have the potential to enhance breast cancer risk.

The degree to which soy is processed will affect the estrogenic properties of products containing a constant amount of genistein[19]. Helferish *et al*.[21] evaluated the ability of various soy products, including soy flour, crude extracts of soy, a mixture of isoflavone and pure genistin, to affect the growth of MCF-7 cells implanted into ovariectomized athymic mice. In animals consuming soy extracts, mixed isoflavones or genistein alone, tumor growth was stimulated compared with those consuming a control diet devoid of soy, while tumors in soy flour-fed animals remained the same in size as that prior to the intervention. Enriched products, like isoflavone, soy protein and soy isolate, have lost some of the beneficially bioactive components and may not have the same healthy benefits as soy foods. Kim *et al*.[22] also compared the influence of diets containing genistein and soy extract on the growth of estrogen-independent human breast cancer cells (MDA-MB-231) that were implanted into female BALB/c mice. It was found that soy extract was more potent than genistein in inhibiting tumor growth, presumably resulting from the synergistic effect of the various bioactive components in the soy extract. It is also suggested that non-ER-mediated actions of isoflavones are anti-proliferative at higher concentrations.

During the past ten years, epidemiological studies of the association between soy exposure and breast cancer risk have used different measures, such as the exposure differences, and there is substantial variation in the percentage of the populations who consume soy and the amount they consume, which renders inconsistent the quantification of the intake and levels of soy foods, soy proteins, soy isolates and dietary isoflavones. This limitation leads to the confusing results regarding this association[2]. At the same time, the research investigating the relationship between soy intake and breast cancer risk has focused increasingly on the anti-cancer mechanism of estrogen dependent and independent effects of isoflavone. Dietary isoflavone level has been investigated in epidemiological studies since 2001. So far, few studies have been conducted on the quantities of isoflavone intake in populations[22]-[25]. No research has been conducted in women with long-term consumption of such highly-processed soy supplements. In the present study, data from the USDA Food and Nutrition Information Center are applied for assessment, because of lack of an authoritative source of food composition database about isoflavone in the country[27]. Due to lack of authoritative data and methods for measuring soy isoflavone, exact interpretation of the summary statistics of this association has been tempered in a way.

The results from our study obviously conflict with the studies reporting that soy-enriched food intake can reduce breast cancer risk. However, the incidence of breast cancer for Asian women was lower due to high consumption of soy food. This is mainly related to healthy life style and direct intake of soy food rather than soy substitutes. Compared with soy-based food like tofu, highly purified dietary soy isoflavone extracts or a single component may stimulate the growth of MCF-7 xenografts. This suggests that some bioactive materials in soy may affect the metabolism of isoflavone *in vivo* to inhibit estrogendependent mammary tumorigenesis and the growth of ER-positive human breast cancer xenografts. In addition, Asian women are more physically active, drink less alcohol, have children earlier, and their entire diet is different from Western women, which all decrease their breast cancer risk. Eating highly refined components of soy can have very different biological effect than eating tofu or drinking soymilk.

Despite the fact that the biological effects are not yet clearly verified, isoflavone supplements have been consumed by healthy women to relieve menopausal symptoms[28],[29]. Recommendations of treatment with high dose of isoflavone supplement for women with a high risk of breast cancer or those who have survived the disease are doubtful and the controversy continues.
